# Effect of Topical Travoprost 0.004% Drops on Central Corneal Thickness in Patients With Primary Open-Angle Glaucoma

**DOI:** 10.7759/cureus.90802

**Published:** 2025-08-23

**Authors:** Marwa Mukhtar, Fahad Zafar, Faizan Luqman, Hafsa Bibi, Haider Sarfaraz

**Affiliations:** 1 Ophthalmology, Medical Teaching Institution (MTI) Ayub Teaching Hospital, Abbottabad, PAK; 2 Ophthalmology, Medical Teaching Institution (MTI) Khyber Teaching Hospital, Peshawar, PAK

**Keywords:** central corneal thickness (cct), intraocular pressure, optic neuropathy, primary open-angle glaucoma, travoprost

## Abstract

Introduction

Glaucoma refers to a spectrum of progressive optic neuropathies that damage the retinal ganglion cells and retinal nerve fiber layer, resulting in characteristic changes in the optic nerve head. It is typically associated with intraocular pressure (IOP)-related damage to the optic nerve head. Recent studies have indicated that reduced central corneal thickness (CCT) is a significant risk factor for open-angle glaucoma. Some studies have also documented a notable reduction in CCT in glaucomatous eyes treated with prostaglandin F2α. We aimed to determine the frequency of decreased CCT with topical travoprost 0.004% drops in patients with primary open-angle glaucoma.

Materials and methods

A descriptive case series of primary open-angle glaucoma patients was performed in the Department of Ophthalmology, Khyber Teaching Hospital, Peshawar, Pakistan, from November 1, 2021, to May 1, 2022. A total of 117 patients of both genders with primary open-angle glaucoma were included in the study. All patients were prescribed topical travoprost 0.004% once daily in the evening in one or both eyes as first-line medication. All patients were followed, and CCT was measured after 10 weeks using ultrasound pachymetry, and CCT was noted. Data was analyzed using SPSS Statistics version 22 (IBM Corp. Released 2013. IBM SPSS Statistics for Windows, Version 22.0. Armonk, NY: IBM Corp.). Decreased CCT was stratified by age, gender, and baseline CCT. The post-stratification chi-square test was applied, and a p-value of <0.05 was considered statistically significant.

Results

In this study, 63 (53.8%) participants were male and 54 (46.2%) were female. The age range included in the study was from 18 to 70 years, with a mean age of 56.794 ± 7.06 years and a mean baseline CCT of 542.059 ± 7.25 µm. Decreased CCT was observed in seven (6%) patients.

Conclusions

Decreased CCT (30 µm or more) was observed in seven (6%) patients. Early CCT reduction with travoprost is usually subclinical, with only 6% of eyes showing ≥30 µm decrease. Although generally mild, such changes may influence IOP readings and glaucoma management, warranting further long-term study of prostaglandin effects on the cornea.

## Introduction

Glaucoma refers to a group of progressive optic neuropathies that damage the retinal ganglion cells and retinal nerve fiber layer, resulting in characteristic changes in the optic nerve head [1]. It is typically associated with intraocular pressure (IOP)-related damage to the optic nerve head [2].

Worldwide, glaucoma is among the primary causes of irreversible blindness and significantly impacts quality of life [3]. Factors that increase risk include old age, genetics, myopia, Black race, family history, smoking, systemic vascular disease, migraine, use of systemic or topical steroids, obstructive sleep apnea, and, most importantly, elevated IOP [4-6].

Recent studies have highlighted the significance of central corneal thickness (CCT) in relation to multiple ocular diseases. Notably, decreased CCT has been recognized as a significant risk factor for open-angle glaucoma [7]. A study was conducted by Hansen and Ehlers, which demonstrated a positive linear correlation between CCT and IOP [8]. A more recent study suggested a correction factor of 2.5 mmHg for each 50 μm change in CCT [9].

The preferred first-line treatment of glaucoma in the developed world is prostaglandin F2α analogues [10]. This is primarily due to their effectiveness in reducing IOP and the once-daily dose. Among the well-documented adverse effects of prostaglandin derivatives are conjunctival hyperemia, hypertrichosis of eyelashes, and increased iris pigmentation [10]. A few studies have observed a notable reduction in CCT in glaucoma patients following treatment with prostaglandin F2α analogues [11].

A study by Schlote et al. has shown that the frequency of decreased CCT was 5.1% after treatment with topical travoprost 0.004% drops in patients with primary open-angle glaucoma [12]. There is no such study done before in our local population. Therefore, this study was conducted to evaluate the effect of travoprost 0.004%, a prostaglandin F2α analogue, on CCT in patients with primary open-angle glaucoma presenting to Khyber Teaching Hospital, Peshawar. The results of this study produced local evidence on this subject.

## Materials and methods

A descriptive case series of primary open-angle glaucoma patients was performed in the Department of Ophthalmology, Khyber Teaching Hospital, Peshawar, Pakistan, from November 1, 2021, to May 1, 2022.

Primary open-angle glaucoma was defined as a patient with a glaucomatous visual field defect and glaucomatous optic neuropathy (vertical cup-disk ratio ≥0.7 or asymmetry between eyes ≥0.2 or minimum rim width <0.1) by ocular examination. Decreased CCT was defined as a reduction in CCT >30 μm from baseline after 10 weeks of treatment with topical travoprost 0.004% drops on ultrasound pachymetry.

Ethical approval for the study was obtained from the Ethics Committee of Khyber Teaching Hospital, endorsed by the Research Evaluation Unit of the College of Physicians and Surgeons Pakistan (approval number: CPSP/REU/OPL-2020-020-2210). Informed consent was taken from all the patients included in the study.

A total of 117 patients of both genders and an age range between 18 and 70 years, with primary open-angle glaucoma, were included in the study. Patients who had prior raised IOP treatment, refractive or intraocular surgery, corneal abnormalities, fundus changes preventing reliable optic disc evaluation, and a history of metabolic and neurological disorders were excluded from the study.

A consecutive non-probability sampling technique was adopted for this study. Sample size was calculated using the WHO sample size software, with a 95% confidence interval, 4% margin of error, and an expected decrease in CCT of 5.1% after treatment with topical travoprost 0.004% drops in patients with primary open-angle glaucoma [12].

Basic demographics like age, gender, and baseline CCT were noted. All patients were prescribed topical travoprost 0.004% once daily in the evening in one or both eyes as first-line medication. All patients were followed, and CCT was measured after 10 weeks on ultrasound pachymetry, and a decrease in CCT was noted.

Data was analyzed using SPSS Statistics version 22 (IBM Corp. Released 2013. IBM SPSS Statistics for Windows, Version 22.0. Armonk, NY: IBM Corp.). Mean and standard deviation (mean ± SD) were presented for quantitative variables like age and baseline CCT. For categorical variables like gender and reduced CCT, frequencies and percentages were calculated. Decreased CCT was stratified by age, gender, and baseline CCT. Following stratification, the chi-square test was applied, and a p-value of less than 0.05 was considered statistically significant.

## Results

The study included patients aged between 18 and 70 years with a mean age of 56.794 ± 7.06 years, and a mean baseline CCT of 542.059 ± 7.25 µm, as shown in Table 1.

**Table 1:** Mean ± SD of patients according to age and baseline CCT (n=117) CCT: central corneal thickness, SD: standard deviation

Parameter	Mean ± SD
Age (years)	56.794 ± 7.06
Baseline CCT (µm)	542.059 ± 7.25

The number of male patients was 63 (53.8%) and the number of female patients was 54 (46.2%), as shown in Table 2.

**Table 2:** Frequency and percentage of patients according to gender (n=117)

Gender	Frequency	Percentage
Male	63	53.8%
Female	54	46.2%
Total	117	100%

Decreased CCT was observed in seven (6%) patients, as shown in Table 3.

**Table 3:** Frequency and percentage of patients according to decreased CCT (n=117) CCT: central corneal thickness

Decreased CCT	Frequency	Percentage
Yes	7	6%
No	110	94%
Total	117	100%

Stratification of decreased CCT with respect to age, baseline CCT, and gender is shown in Tables 4-6, respectively.

**Table 4:** Stratification of decreased CCT with respect to age (n=117) CCT: central corneal thickness Chi-square value: 0.078

Age (years)	Decreased CCT		p-value
	Yes	No	
18-45	0 (0%)	12 (100%)	0.779
46-70	7 (6.7%)	98 (93.3%)	
Total	7 (6%)	110 (94%)	

**Table 5:** Stratification of decreased CCT with respect to baseline CCT (n=117) CCT: central corneal thickness Chi-square value: 1.631

Baseline CCT (µm)	Decreased CCT		p-value
	Yes	No	
≤540	0 (0%)	33 (100%)	0.201
>540	7 (8.3%)	77 (91.7%)	
Total	7 (6%)	110 (94%)	

**Table 6:** Stratification of decreased CCT with respect to gender (n=117) CCT: central corneal thickness Chi-square value: 0.326

Gender	Decreased CCT		p-value
	Yes	No	
Male	5 (7.9%)	58 (92.1%)	0.567
Female	2 (3.7%)	52 (96.3%)	
Total	7 (6%)	110 (94%)	

## Discussion

Prostaglandins are a group of molecules that are present in most tissues and organs in the body. They are enzymatically derived from fatty acids and all consist of 20 carbon atoms, including a five-carbon ring. They serve a variety of functions, including inflammation, muscle constriction, and platelet aggregation. This wide range of functions is regulated by binding to different types of prostaglandin receptors. These receptors are transmembrane, G-protein-coupled receptors. Like latanoprost and bimatoprost, travoprost is a synthetic analogue of prostaglandin F2α [12].

The interaction between ocular-hypotensive drugs and CCT is of particular interest due to their potential impact on applanation tonometry in the follow-up of glaucoma patients. Evidence from experimental studies has shown a possible interaction between prostaglandin analogues and various ocular tissues, including keratocytes, resulting in structural and compositional changes in the extracellular matrix [12].

In our study, only a small proportion of eyes (6%) showed a decrease in CCT greater than 30 μm after 10 weeks of treatment with the prostaglandin F2α analogue, travoprost 0.004%. Our study results are consistent with the research conducted by Schlote et al., which demonstrated that the frequency of decreased CCT was 5.1% in primary open-angle glaucoma patients after treatment with topical travoprost 0.004% [12].

Therefore, measurement of CCT again after 10 weeks of travoprost therapy may be helpful in progressive glaucoma to establish a more accurate target pressure. No association was found between reduction in CCT and demographic parameters (age, gender), which is consistent with the results of a study done by Viestenz et al. [13].

Prostaglandin analogues are likely to alter the CCT within a few months of therapy. A prospective study involving 34 patients who were treated with latanoprost, bimatoprost, and travoprost for a period of four weeks demonstrated a small but statistically significant reduction in mean CCT (0.6 ± 1.3%) after bimatoprost use [14]. However, travoprost and latanoprost did not lead to statistically significant changes. In a study by Sen et al., there was a significant reduction in CCT following monotherapy with latanoprost or bimatoprost in 188 eyes of 94 patients at six, 12, and 24 months. The mean CCT reduction was 1.9 ± 2.4% with latanoprost and 2.8 ± 1.8% with bimatoprost, compared to the baseline CCT [15]. However, no statistically significant difference was observed between the groups treated with latanoprost and bimatoprost. Another prospective study revealed that monotherapy with latanoprost, travoprost, and bimatoprost over eight weeks led to a measurable decrease in CCT. All three treatment groups showed a statistically significant mean reduction in CCT of 4-6 μm, whereas no such change was demonstrated in the unmedicated control group [16]. The findings of our study expand the existing literature and contribute to the ongoing and important discussion regarding the effect of prostaglandin analogues on the cornea.

Our study was conducted in a routine clinical setup and, therefore, had certain limitations. Our study showed a nonlinear CCT reduction that occurred over a period of 10 weeks of treatment. Further research is needed to explore if prolonged treatment duration leads to greater CCT reduction and whether it has a significant effect on applanation tonometry. Moreover, it is not yet known whether prostaglandin-induced changes in CCT may also induce changes in the biomechanical properties of the cornea, as observed in patients with corneal disorders or following refractive laser procedures [17].

## Conclusions

So far, CCT reduction observed during the early phase (first 10 weeks) of travoprost therapy seems to be a subclinical finding in most eyes. Only a small percentage of eyes (6%) showed CCT reduction of 30 μm or more. Although such changes are generally mild, they may affect IOP readings and have implications for glaucoma management. Further research is needed to understand better the long-term impact of prostaglandin analogues on the cornea.
